# Supplementary material for: HOMA Index, Vitamin D Levels, Body Composition and Cardiorespiratory Fitness in Juvenile Obesity: Data from the CHILT III Programme, Cologne
Source: Int J Environ Res Public Health. 2022 Feb 20;19(4):2442. doi: 10.3390/ijerph19042442 (PMC8872273; doi:10.3390/ijerph19042442)
Supplement: Supplementary file 1 [file ijerph-19-02442-s001.zip › ijerph-1557707-supplementary.pdf]

Table S1: Pearson correlation coefficients between the 25(OH)D concentration and body composition, physical fitness and HOMA Index.

**Variable**	**BMI SDS**		**Body fat (kg)**		**Body fat (%)**		**Absolute cardiorespiratory fitness (W)**		**Relative cardiorespiratory fitness (W/kg)**		**Vitamin D (ng/ml)**		**HOMA Index**	
	**P-value (n)**	**r**	**P-value (n)**	**r**	**P-value (n)**	**r**	**P-value (n)**	**r**	**P-value (n)**	**r**	**P-value (n)**	**r**	**P-value (n)**	r
Age (years)	.495 (n=144)	-0.057	**<.001**† (n=137)	0.614	**.003**† (n=137)	0.256	**<.001**† (n=135)	0.688	.435 (n=135)	0.068	.236 (n=147)	-0.098	**<.001**† (n=147)	0.270
Height (m)	.923 (n=144)	-0.008	**<.001**† (n=137)	0.698	**<.001**† (n=137)	0.329	**<.001**† (n=135)	0.766	.449 (n=135)	0.066	.182 (n=144)	-0.112	**<.001**† (n=144)	0.393
Weight (kg)	**<.001**† (n=144)	0.540	**<.001**† (n=137)	0.976	**<.001**† (n=137)	0.599	**<.001**† (n=135)	0.579	**<.001**† (n=135)	-0.308	**.010**† (n=144)	-0.213	**<.001**† (n=144)	0.459
Waist circumference (cm)	**<.001**† (n=136)	0.580	**<.001**† (n=136)	0.830	**<.001**† (n=136)	0.607	**.002**† (n=135)	0.265	**<.001**† (n=135)	-0.453	**<.001**† (n=136)	-0-308	**<.001**† (n=136)	0.416
BMI (kg/m^2)	**<.001**† (n=144)	0.814	**<.001**† (n=137)	0.891	**<.001**† (n=137)	0.632	**.003**† (n=135)	0.257	**<.001**† (n=135)	-0.505	**.008**† (n=144)	-0.221	**<.001**† (n=144)	0.359
BMI SDS			**<.001**† (n=137)	0.589	**<.001**† (n=137)	0.540	.384 (n=135)	-0.076	**<.001**† (n=135)	-0.581	**.024**† (n=144)	-0.188	**.005**† (n=144)	0.233
Body fat (kg)	**<.001**† (n=137)	0.589			**<.001**† (n=137)	0.743	**<.001**† (n=135)	0.497	**<.001**† (n=135)	-0.364	**.001**† (n=137)	-0.270	**<.001**† (n=137)	0.435
Body fat (%)	**<.001**† (n=137)	0.540	**<.001**† (n=137)	0.743			.099 (n=135)	0.143	**<.001**† (n=135)	-0.402	**.002**† (n=137)	-0.266	**<.001**† (n=137)	0.291
Absolute physical fitness (W)	.384 (n=135)	-0.076	**<.001**† (n=135)	0.497	.099 (n=135)	0.143			**<.001**† (n=135)	0.508	.912 (n=135)	0.010	**.007**† (n=135)	0.233
Relative physical fitness (W/kg)	**<.001**† (n=135)	-0.581	**<.001**† (n=135)	-0.364	**<.001**† (n=135)	-0.402	**<.001**† (n=135)	0.508			**.008**† (n=135)	0.227	.111 (n=135)	-0.138
Vitamin D (ng/ml)	**.024**† (n=144)	-0.188	**.001**† (n=137)	-0.270	**.002**† (n=137)	-0.266	.912 (n=135)	0.010	**.008**† (n=135)	0.227			**.003**† (n=147)	-0.244
Blood glucose (mg/dl)	**.025**† (n=144**)**	-0.186	.253 (n=137)	0.098	.666 (n=137)	0.037	**.041**† (n=135)	0.176	.565 (n=135)	0.050	.174 (n=147)	0.113		
Insulin (μU/ml)	**<.001**† (n=144)	0.273	**<.001**† (n=137)	0.434	**<.001**† (n=137)	0.295	**.014**† (n=135)	0.212	.102 (n=135)	-0.141	**.006**† (n=147)	-0.226		
HOMA index	**.005**† n=(144**)**	0.233	**<.001**† (n=137**)**	0.435	**<.001**† (n=137)	0.291	**.007**† (n=135)	0.233	.111 (n=135)	-0.138	**.003**† (n=147)	-0.244		

The data are presented as the mean ± SD; BMI = body mass index; SDS = standard deviation score; HOMA = homeostasis model assessment; p-values calculated with the †*Pearson-correlation*
